# Study on the effect of *Portulaca oleracea* L. extract on chronic ulcerative colitis in rats induced by allogeneic antigen and acetic acid

**DOI:** 10.1590/acb412126

**Published:** 2026-05-11

**Authors:** Yan Wu, Fang Zhao, Juan Zhao, Rong-Yao Ruan, Fei-Yan Wu, Yusheng Xu

**Affiliations:** 1 Zhejiang Chinese Medical University – Zhejiang – China.; 2 Lishui Second People’s Hospital – Pharmacy Department – Zhejiang – China.; 3 Pu’er University – School of Tea and Coffee – Yunnan – China.; 4 Pu’er University – Yunnan International Joint Laboratory of Digital Conservation and Germplasm Innovation and Application – Laos Tea Resources – Yunnan – China.; 5 Qingyuan County Maternal and Child Health and Family Planning Service Center – Zhejiang – China.

**Keywords:** *Portulaca oleracea* L., Colitis, Ulcerative, Inflammation, Acetic Acid

## Abstract

**Purpose:**

To investigate the effect and mechanism of Portulaca oleracea L. (POL) extract on acetic acid and alloantigen-induced chronic ulcerative colitis in rats.

**Methods:**

Ulcerative colitis was induced in rats by sensitization with rabbit colon antigen and acetic acid. Rats received oral sulfasalazine, intestine-quieting tablet, or POL (high, medium, or low dose) for 14 days. Disease activity index (DAI), colonic mucosal damage index (CMDI), and histological score (HS) were assessed. Serum levels of interleukin (IL)-4, IL-8, IL-17, C-reactive protein (CRP), immunoglobulin G (IgG), and interferon-γ (IFN-γ); colonic levels of myeloperoxidase (MPO), epidermal growth factor (EGF), and tumor necrosis factor (TNF)-α; serum hemolysin (by immunoturbidimetry); splenic T lymphocyte subsets (by flow cytometry); and serum IgG, IgM, IgA, C3, and C4 were measured.

**Results:**

Compared with the model group, POL significantly reduced DAI, CMDI, and HS scores in a dose-dependent manner—most markedly in the high-dose group (*p* < 0.01). POL also significantly decreased serum IL-8, IL-17, CRP, IFN-γ, and IgG, and colonic TNF-α and MPO (*p* < 0.05 or *p* < 0.01), while increasing serum IL-4 and colonic EGF (*p* < 0.01). All POL dosage groups significantly increased hemolysin, IgM, and CD4+CD25+/CD4+ levels in rats (*p* < 0.05 or *p* < 0.01), while decreasing serum IgG, IgA, complement C3/C4, CD4+/CD8+, and IL-17+ CD4+ (*p* < 0.05 or *p* < 0.01).

**Conclusion:**

The POL extract exerts therapeutic effects in chronic ulcerative colitis rats, likely by downregulating pro-inflammatory mediators—including IL-8, IL-17, CRP, IFN-γ, TNF-α, and MPO—, as well as serum IgG, IgA, C3/C4, CD4+/CD8+, and IL-17+ CD4+; and by upregulating anti-inflammatory/repair factors—including IL-4, EGF, lysozyme, IgM, and CD4+CD25+/CD4+. These findings support the clinical potential of POL for ulcerative colitis treatment.

## Introduction

Ulcerative colitis (UC) and Crohn’s disease fall under the category of inflammatory bowel diseases, which are a common chronic non-specific intestinal disorder primarily affecting the mucosa and submucosa of the colon and rectum, presenting with both segmental and diffuse distribution^1^. Clinical manifestations of UC often include abdominal pain, diarrhea, urgency, and tenesmus, as well as mucous bloody stools. Furthermore, it may be accompanied by severe complications. UC is characterized by a high mortality rate in acute onset cases, a high risk of cancer transformation in chronic persistent cases, and a tendency for recurrent episodes. It has been defined as a precancerous disease and classified as a globally refractory disease by the World Health Organization^2,3^.

With the significant changes in people’s lifestyles, dietary habits, and environment, the spectrum of diseases has also changed. The increasing incidence of UC year by year is a reflection of these changes. Based on the statistics from several hospitals in China, it is estimated that the prevalence of UC in China is 11.6 per 100,000 (possibly underestimated). In Western countries, the incidence rate ranges from 10 to 20 per 100,000. and the prevalence rate ranges from 100 to 200 per 100,000. The onset age of UC is between 15–40 years old, with a possible second peak between ages 50–80. There is no significant difference in incidence between men and women, and there is a familial clustering^4,5^.

The etiology of UC is complex, and its pathogenesis is currently not fully understood. It is widely believed that the onset of UC is closely related to genetic factors, environmental influences, infections, and immune responses^6-9^. Currently, there are eight main categories of Western medicine used in the clinical treatment of UC: 5-aminosalicylic acid, corticosteroids, immunosuppressants, novel immunomodulators, anticoagulants, antibiotics, biologics, and probiotics^10^. Although there is currently a wide variety of clinical treatment drugs available, there is still no specific effective treatment plan and medication for this disease. Moreover, long-term use of non-steroidal anti-inflammatory drugs and corticosteroids can lead to numerous adverse reactions. Therefore, it is particularly important to seek out effective methods and medications for treating this disease.

Traditional Chinese medicine (TCM) is a treasure of the Chinese nation and a crystallization of the wisdom of TCM practitioners. According to TCM, UC often occurs due to congenital deficiency, weakness of the spleen and stomach, invasion of damp-heat pathogen, overconsumption of rich and greasy food leading to damp-heat accumulation, or transformation of

cold-damp into heat invading the intestines. This results in poor circulation of qi, obstruction in descending function, stasis of blood circulation, putrefaction and decay of flesh due to injury to the network vessels, ultimately forming internal ulcers. The treatment of TCM mainly involves the internal use of Chinese herbal medicine, supplemented by enema therapy. Chinese herbal medicine can be taken for a long time with minimal side effects. The use of TCM in the prevention and treatment of UC has shown a clinical overall effective rate of 92.1%, which is significantly higher than that of chemical drugs^11,12^. Therefore, traditional Chinese medicine has significant clinical application value in the treatment of UC.

*Portulaca oleracea* L. (POL) was first documented in the “Compendium of Materia Medica” during the Southern and Northern Dynasties of China^13^. In 2002, the National Health Commission of the People’s Republic of China (Weifa Jianfa No. 51) included *P. oleracea* L. in its list of homologous medicinal and food items. According to traditional Chinese pharmaceutical texts, *P. oleracea* L. is characterized by a sour taste and cold nature. It is associated with the large intestine and liver meridians. This plant exhibits properties that cool blood, detoxify, eliminate dampness, alleviate stranguria, and stop dysentery. It is commonly used internally to treat conditions such as bacterial dysentery, acute gastroenteritis, acute appendicitis, leucorrhea, among others^14,15^. Furthermore, they have shown good therapeutic effects on acute UC induced by oxazolone in rats and chronic UC induced by 2,4-dinitrochlorobenzene combined with acetic acid in rats^16,17^.

In this study, a UC model was established using the antigen sensitization combined with acetic acid induction method. The aim was to create an animal model that closely resembles the pathogenesis of human UC. The therapeutic effect of POL on UC rats was observed by administering it orally and evaluating changes in general condition, body weight, disease activity index (DAI) score, organ index, colon colonic mucosal damage index (CMDI) score, splenic T lymphocyte subsets, serum antibodies, colonic histopathology, and biochemical indicators. The mechanism of action of POL on antigen sensitization combined with acetic acid-induced UC in BALB/c rats was investigated with the goal of providing sufficient experimental evidence for the development of POL as a drug for treating UC.

## Methods

### Experimental animals

Health specific-pathogen free level Sprague Dawley rats, 100 in total, with a body weight of 180–220 g, half male and half female [Hunan Sleek Jinda Experimental Animal Co., Ltd., License No.: SCXK (Xiang) 2023-0004], were used in the study. Rat feed and corn cob bedding were purchased from Chengdu Dashuo Experimental Animal Co., Ltd., Batch No.: 20230701. All experimental studies complied with the relevant animal research guidelines of the Chinese Ethics Committee.

### Instruments and equipment

For the experiment, we used: desktop high-speed refrigerated centrifuge (Shanghai Centrifuge Mechanical Research Institute, Model: TL-16R); electronic analytical balance (Mettler-Toledo Instruments Co., Ltd., Model: ML204/02); ultraviolet-visible spectrophotometer (Beijing Puxi General Instruments Co., Ltd., Model: T6 New Century); enzyme labeling instrument (Austria Anteus Company, Model: 201); and freeze dryer (GOLD-SIM Company, Model: FD8-4a).

### Experimental drugs and reagents

We used in the experiment: POL extract; sulfamethoxypyridazine (SASP) (Shanghai Xinyi Tianping Pharmaceutical Co., Ltd., Batch number: 09160503); ChangYanNing (CYN) (Jiangxi Tianshi Kangyi Pharmaceutical Co., Ltd., Batch Number: 220303).

The complete Freund’s adjuvant (Sigma, batch number: SLBK1733), tumor necrosis factor-α (TNF-α) enzyme-linked immunosorbent assay (ELISA) kit (batch number: 160927-102a), interleukin (IL)-4 ELISA kit (batch number: 160927-002b), IL-8 ELISA kit (batch number: 161025-008b), and interferon-γ (IFN-γ) ELISA kit (batch number: 161025-101b) were all provided by Xinbosheng QuantiCyto Biotechnology Co., Ltd. The C-reactive protein (CRP) assay kit (Lot No: 20221215), the immunoglobulin G (IgG) assay kit for rats (Lot No: 20161101), the IL-17 assay kit for rats (Lot No: 20161228), the myeloperoxidase (MPO) assay kit for rats (Lot No: 20221022), the epidermal growth factor (EGF) assay kit for rats (Lot No: 20221228), and the C3 assay kit for rats (Lot No: 20220824), as well as the C4 assay kit for rats (Lot No: 20220824), were all provided by Nanjing Jiancheng Bioengineering Institute.

### Experimental method

#### Model establishment

Ten rats (half male and half female) were randomly selected as the control group, while the remaining rats were used for UC modeling. Six hundred mg of freeze-dried powder was prepared from rabbit colon using a precision balance and dissolved in 3.75 mL of physiological saline. After complete dissolution, 3.75 mL of complete Freund’s adjuvant was added, vortex to mix well, and the emulsion antigen reagent was obtained (used immediately after preparation). On days 1, 8, 15, 22, and 29, a total of 0.1 mL of antigen emulsion was injected into the left footpad, right toe, inguinal region, subcutaneously on the back, and intraperitoneally in rats. After the final injection of antigen emulsion (without complete Freund’s adjuvant), fasting without water for 24 hours was implemented. Rats were then given a rectal enema of 1 mL of 5% acetic acid and retained for 15 seconds. This was followed by irrigation with 4 mL of normal saline and another rectal enema with 1 mL of antigen emulsion. After retaining for an additional 30 minutes, rats were rinsed with 4 mL of normal saline to complete the modeling process. The control group should follow the same procedure with the reagent being replaced by physiological saline solution.

#### Grouping and administration

On the second day after the completion of modeling, reference was made to Hamamoto et al.^18^ for scoring the DAI. The severity of inflammation is classified as follows:

- 0–3 points: very mild;
- 4–6 points: mild;
- 7–9 points: moderate;
- 10–12 points: severe.

Rats with mild and very mild inflammation according to DAI were excluded. The remaining 60 rats were stratified by the degree of inflammation and randomly divided into model group, SASP group (300 mg/kg), CYN group (300 mg/kg), POL high, medium, and low dose groups (200, 100, and 50 mg/kg), with 10 rats in each group equally divided between male and female. Except for the normal control group and model group which were given physiological saline by gavage, the remaining groups were given corresponding volumes of drugs by gavage once a day for continuous administration for 14 days.

#### Observation indicators and methods

During the observation and recording of the administration period, changes in the external behaviors such as fur color, diet, urination, and defecation of each group of rats were noted. Additionally, DAI scores were assessed on days 1, 4, 7, 10, and 14 of administration (Table 1). After the last administration, fasting but not water restriction for 24 hours was applied to anesthetized rats. Blood samples were collected from the abdominal aorta and allowed to clot at 4°C, followed by centrifugation at 3,000 rpm for 10 minutes to obtain serum. The levels of IL-4, IL-8, IL-17, CRP, IFN-γ, and IgG in rat serum were measured according to the instructions of the reagent kit.

**–**

**Table 1:** Evaluation of disease activity index (DIA).

Score of DIA	Stool consistency	Occult blood test	Weight loss (%)
0	Normal	Negative (-)	< 1
1	Normal-sparse stool	Weak positive (+)	1–5
2	Sparse stool	Positive (++)	5–10
3	Sparse stool-diarrhea	Strong positive (+++)	10–15
4	Diarrhea	Bloody stool	≥ 15

Source: Elaborated by the authors.

After euthanizing the rats, the liver, spleen, lungs, and colon were removed and weighed to calculate organ indices. The colon was dissected along the mesentery for measurement of length and width, as well as for assessment of CMDI scores (Table 2). Colonic lesions were dissected and 10% tissue homogenates were prepared, followed by centrifugation to obtain the supernatant for the measurement of EGF, MPO, and TNF-α levels. The remaining colon was fixed in 10% neutral formalin for histopathological sectioning and histological score (HS) analysis^19^ (Table 3).

**Table 2:** Colonic mucosal damage index (CMDI) scoring criteria.

Score	The mucous membrane of the colon
0	No damage
1	Mild hyperemia and edema, smooth surface, no erosion or ulcer
2	Hyperemia and edema, rough mucous membrane, granular sensation, erosion or intestinal adhesion
3	Severe hyperemia and edema, necrosis and ulcer formation on the surface, thickening of intestinal wall or necrosis and inflammatory polyps on the surface
4	Severe hyperemia and edema, mucosal necrosis and ulcer formation, whole intestinal wall necrosis, death caused by toxic megacolon

Source: Elaborated by the authors.

**Table 3:** Scoring criteria of histopathology<tfn>*</tfn>.

Score	Epithelial cell	Degree of inflammatory cell infiltration
0	Normal form	No infiltration
1	Loss of a small number of goblet cells	Infiltrate into the basal layer of the crypt
2	Massive loss of goblet cells	Infiltrate into the muscular layer of the mucosa
3	Loss of a small number of crypt cells	Infiltrate into the muscular layer of the mucosa, accompanied by mucosal thickening and obvious edema
4	Massive loss of crypt cells	Infiltrate into the submucosa

* Colonic histological score = epithelial cell score + inflammatory cell infiltration score.

Source: Elaborated by the authors.

#### Detection of hemolysin production levels in rats

At the 7th, 9th, and 11th day, intraperitoneal injection of 0.2 mL of 20% sheep red blood cells was administered for sensitization. Blood was collected 1 hour after the last dose, centrifuged at 2,000 rpm for 10 minutes to obtain the supernatant serum. The serum was then incubated at 56°C in a water bath for 30 minutes to inactivate complement before titration of hemolytic complement activity. After diluting the serum at the corresponding multiple, it was placed in an Eppendorf tube, then we added 0.5 mL of 5% sheep red blood cells. The guinea pig serum was diluted by 1 mL, and we used physiological saline instead of rat serum in the blank control tube.

All test tubes were incubated at 37℃ in a water bath for 10 minutes to maintain a constant temperature, then removed and placed in an ice bath to terminate the reaction. The supernatant (1 mL) was collected after centrifugation at 2,000 rpm for 10 minutes, and mixed with 3 mL of the Dacie reagent in a tube. Simultaneously, 0.25 mL of sheep red blood cell suspension was mixed thoroughly with 4 mL of the Dacie reagent in another tube and allowed to stand for 10 minutes. A blank control group was set up, and the wavelength was measured under the same conditions, then the hemolysis value was calculated (Eq. 1):


(1) HC50=(Sampleabsorbancevalue/AbsorbanceathalfhemolysisofSRBC)×Dilutionfactor

#### Detection of T cell subpopulation changes in the spleen by flow cytometry

To isolate the single splenic cells, fresh spleens from rats were placed on a 200-mesh cell strainer, and ground into a single-cell suspension using a 5-mL syringe plunger. The cell suspension was collected after centrifugation, the supernatant was discarded, and 1 mL of red blood cell lysis buffer was added. After complete lysis of the red blood cells, 4 mL of 3% FCS-PBS was added for washing twice before discarding the supernatant. The isolated splenic single cells were resuspended in RPMI-1640 complete culture medium. The cell suspension was stained with 0.4% trypan blue staining solution for cell counting, and the cell concentration was adjusted to 1×10^7^ cells/mL.

To determine the lymphocyte subset, 100 μL of splenocyte suspension (1×10^6^ cells) was added to a flow tube, followed by the addition of corresponding mixed fluorescent antibodies. The sample was then analyzed using FACS Calibur to determine the proportion of T lymphocytes (CD3+) in individual splenic cells. Specifically, the proportions of Th cells (CD3+ CD4+ CD8-), Tc cells (CD3+ CD4- CD8+), and Treg cells (CD3+ CD4+ CD25+) within the splenic T lymphocyte population were assessed.

### Statistical method

Statistical description and statistical analysis of data were performed using the Statistical Package for the Social Sciences 21.0 and GraphPad Prism 5.0. Measurement data were expressed as mean ± standard deviation, and t-test and one-way analysis of variance were used for data that conformed to normal distribution and homogeneous variance, and data that did not conform to normal distribution were used rank sum test. Repeated measures analysis of variance and one-way analysis of variance were used for continuous data, and LSD test was used for pairwise comparison between groups. *P* < 0.05 was used as the standard for statistically significant differences.

## Results

### General symptoms observation in rats

The normal group of rats had smooth and shiny fur, full of energy, with normal food and water intake. They were agile and active, with stable body weight. Their feces were firm and well-formed. The rats in the model group were lethargic, their body weight dropped sharply, their coats were loose and dull, and they had arched backs, bunching up, bloody or loose stools, and feces adhering to the anus. Rats in the CYN and SASP groups exhibited significantly improved mental function compared to the model group. They showed increased activity, absence of back arching, bunching up, and lethargy, as well as formed feces. Following administration of POL at high (200 mg/kg), medium (100 mg/kg), and low doses (50 mg/kg) to the rats, symptoms such as loose stools and bloody stools were reduced. Additionally, their activity levels increased and their mental status improved.

### Effect of Portulaca oleracea L. on disease activity index scores in ulcerative colitis rats

On the first day post-modeling, the rats exhibited varying degrees of weight loss, diarrhea, and bloody stools. Compared to the control group, the DAI scores of rats in the experimental groups showed a significant increase (*p* < 0.01). There were no statistically significant differences observed between the experimental groups, indicating successful modeling. The DAI score decreased gradually with the increase in treatment time. On the 14th day of administration, compared to the model group, each dose group of POL showed a decreasing trend in DAI score. Among them, the high-dose group exhibited the most significant decrease (*p* < 0.01), which was comparable to the positive control group. The efficacy of drugs SASP and CYN is equivalent (Table 4).

**Table 4:** Effect of *Portulaca oleracea* L. on the disease activity index score of ulcerative colitis rats (n = 10, mean ± standard deviation).

Group	Dose (mg/kg)	1st day	4th day	7th day	10th day	14th day
N	-	1.0 ± 0.0	1.1 ± 0.8	1.2 ± 0.4	1.0 ± 0.0	1.2 ± 0.8
M	-	9.5 ± 1.4**	8.2 ± 1.0**	8.7 ± 0.9**	9.5 ± 1.4**	9.2 ± 1.0**
SASP	300	9.9 ± 1.3**	7.0 ± 1.5**	4.5 ± 1.1** ^△△^	3.7 ± 1.3** ^△△^	2.8 ± 1.5** ^△△^
CYN	300	9.4 ± 1.2**	7.7 ± 1.3**	5.9 ± 0.9** ^△△^	3.4 ± 1.2** ^△△^	2.4 ± 1.3** ^△△^
POL	50	9.3 ± 1.3**	7.0 ± 1.2**	5.2 ± 2.0** ^△△^	3.3 ± 1.3** ^△△^	2.2 ± 0.9** ^△△^
POL	100	9.4 ± 1.1**	7.4 ± 1.0**	6.3 ± 0.8** ^△△ ▲^	3.4 ± 1.1** ^△△^	2.4 ± 1.0** ^△△^
POL	200	9.6 ± 1.4**	7.1 ± 0.9**	5.1 ± 1.1** ^△△^	2.9 ± 1.4** ^△△^	1.8 ± 1.2** ^△△^

N: normal control group; M: model group; SASP: sulfamethoxypyridazine; CYN: ChangYanNing; POL: *Portulaca oleracea* L.; *p < 0.05 compared with the normal control group;

** *p* < 0.01 compared with the normal control group;

^△^ *p* < 0.05 compared with the model group;

*p* < 0.01 compared with the model group;

△△ *p* < 0.05 compared with SASP.

▲ Source: Elaborated by the authors.

### Effect of Portulaca oleracea L. on colon damage in ulcerative colitis rats

The colonic mucosa of the rats in the normal group appeared smooth and free from disease upon naked eye observation. In contrast, when the rats in the model group were longitudinally dissected along the mesentery, their colonic mucosa exhibited significant congestion, edema, ulcers, and erosion, with the most severe lesions located in the rectum. As the damage to the colon increased, the length of the rat’s colon shortened while the width, colon index, and CMDI score increased. In comparison to the normal group, the rats in the model group showed a significant increase in colon index, width, and CMDI score (*p* < 0.01), as well as a significant decrease in length (*p* < 0.01). Compared to the control group, the length of the rats’ colons in each treatment group showed a significant increase (*p* < 0.05 or *p* < 0.01), while the colon index, width, and CMDI score exhibited a significant decrease (*p* < 0.05 or *p* < 0.01). Furthermore, when compared to the SASP group and the high-dose POL group, there was a significant reduction in colon index (*p* < 0.05), with therapeutic effects equivalent to CYN (Table 5).

**Table 5:** Effect of POL on the colon related indicators of ulcerative colitis rats (n = 10, mean ± standard deviation).

Group	Dose (mg/kg)	Colon index (mg/kg)	Length (cm)	Width (cm)	CMDI score (point)
N	-	4.52 ± 0.61	22.18 ± 2.33	0.67 ± 0.10	1.0 ± 0.1
M	-	7.12 ± 0.7**	15.44 ± 3.51**	1.58 ± 0.42**	4.4 ± 1.3**
SASP	300	5.52 ± 0.88* ^△△^	16.37 ± 3.62**	0.66 ± 0.32^△△^	2.1 ± 0.6** ^△△^
CYN	300	4.61 ± 0.65^△△^	17.28 ± 1.24** ^△^	0.73 ± 0.25^△△^	2.0 ± 0.6** ^△△^
POL	50	5.58 ± 0.19* ^△△^	16.64 ± 3.57**	0.98 ± 0.68^△△^	2.6 ± 1.2** ^△△^
POL	100	5.55 ± 0.54* ^△△^	17.404 ± 2.55** ^△^	0.71 ± 0.28^△△^	2.7 ± 1.4** ^△△^
POL	200	4.65 ± 0.67^△△ ▲^	18.09 ± 3.83** ^△^	0.66 ± 0.37^△△^	2.1 ± 1.2** ^△△^

N: normal control group; M: model group; SASP: sulfamethoxypyridazine; CYN: ChangYanNing; POL: *Portulaca oleracea* L.;

* *p* < 0.05 compared with the normal control group;

** *p* < 0.01 compared with the normal control group;

△ *p* < 0.05 compared with the model group;

△△ *p* < 0.01 compared with the model group;

▲ *p* < 0.05 compared with SASP;

Source: Elaborated by the authors.

### Effect of Portulaca oleracea L. on the expression levels of interleukin (IL)-4, IL-8, IL-17, C-reactive protein, interferon-γ, and immunoglobulin G in the serum of ulcerative colitis rats

Compared to the normal group, rats in the model group showed a significant increase in the levels of IL-8, IL-17, CRP, IFN-γ, and IgG in their serum (*p* < 0.01), while the levels of IL-4 were significantly decreased (*p* < 0.01). Compared to the model group, the levels of IL-8, IL-17, CRP, and IFN-γ in each administration group were significantly reduced (*p* < 0.01). Additionally, the levels of IgG in the SASP, CYN, and POL high-dose groups were also significantly reduced (*p* < 0.05 or *p* < 0.01), while the IL-4 content in each administration group increased significantly (*p* < 0.01) (Table 6).

**Table 6:** Effect of *Portulaca oleracea* L. on serum inflammatory factor levels in ulcerative colitis rats (n = 10, mean ± standard deviation).

Group	Dose (mg/kg)	IL-4	IL-8	IL-17	CRP (mg/L)	IFN-γ ( pg/mL)	IgG (mg/mL)
N	-	26.49 ± 4.12	240.51 ± 11.38	14.12 ± 2.56	0.88 ± 0.41	2.80 ± 1.49	9.38 ± 0.91
M	-	8.40 ± 1.49**	601.21 ± 34.33**	43.16 ± 2.44**	4.92 ± 1.06**	11.56 ± 2.52**	15.31 ± 1.79**
SASP	300	19.22 ± 4.12^△△^	255.15 ± 13.68^△△^	15.33 ± 2.16^△△^	1.13 ± 0.22^△△^	6.95 ± 2.06** ^△△^	12.26 ± 0.91** ^△△^
CYN	300	24.17 ± 4.12^△△^	261.35 ± 09.58^△△^	20.71 ± 2.66* ^△△^	1.21 ± 0.21^△△^	7.29 ± 1.99** ^△△^	13.18 ± 0.91** ^△△^
POL	50	23.53 ± 4.12^△△^	242.66 ± 24.58^△△^	15.17 ± 2.22^△△^	1.95 ± 0.60* ^△△^	5.80 ± 2.12** ^△△^	13.64 ± 0.91**
POL	100	23.36 ± 4.12^△△^	260.36 ± 11.58^△△^	15.21 ± 2.36^△△^	1.30 ± 0.11^△△^	6.47 ± 2.55** ^△△^	14.22 ± 0.91**
POL	200	28.54 ± 4.95^△△ ▲^	245.55 ± 65.31^△△^	16.43 ± 3.02^△△^	1.03 ± 0.13^△△^	4.26 ± 1.54* ^△△^	11.98 ± 3.40* ^△^

N: normal control group; M: model group; SASP: sulfamethoxypyridazine; CYN: ChangYanNing; POL: *Portulaca oleracea* L.;

* *p* < 0.05 compared with the normal control group;

** *p* < 0.01 compared with the normal control group;

△ *p* < 0.05 compared with the model group;

△△ *p* < 0.01 compared with the model group;

▲ *p* < 0.05 compared with SASP; IL: interleukin; CRP: C-reactive protein; IFN-γ: interferon-γ; IgG: immunoglobulin G.

Source: Elaborated by the authors.

### Effect of Portulaca oleracea L. on the expression levels of epidermal growth factor, myeloperoxidase, and tumor necrosis factor-α in colon tissue of ulcerative colitis rats

Compared to the control group, the EGF content in the model group of rats showed a significant decrease (*p* < 0.01), while the TNF-α and MPO contents exhibited a significant increase (*p* < 0.01). Compared to the model group, the TNF-α and MPO contents of rats in each treatment group were significantly reduced (*p* < 0.01). The SASP group, CYN group, and POL high and medium dose groups were able to significantly increase the EGF content in tissues (*p* < 0.01), demonstrating a strong dose dependence. In comparison with the positive drug group, the high-dose POL group showed a significant increase in EGF content (*p* < 0.01), and a decrease in MPO and TNF-α content (*p* < 0.05 or *p* < 0.01) (Table 7).

**Table 7:** Effect of POL on biochemical indicators of colon tissue in ulcerative colitis rats (n = 10, mean ± standard deviation).

Group	Dose (mg/kg)	EGF (pg /mL)	MPO (U/Gram tissue wet weight)	TNF-α (pg /mL)
N	-	355.21 ± 24.54	0.18 ± 0.02	556.30 ± 68.49
M	-	197.49 ± 8.21**	0.62 ± 0.07**	878.81 ± 43.03**
SASP	300	258.79 ± 28.09** ^△△^	0.30 ± 0.08** ^△△^	717.69 ± 42.91** ^△△^
CYN	300	245.92 ± 19.56** ^△△^	0.36 ± 0.06** ^△△^	706.30 ± 58.93** ^△△^
POL	50	193.85 ± 35.51** ^▲▲▽▽^	0.44 ± 0.09** ^△△ ▲▲^	713.46 ± 75.77** ^△△^
POL	100	255.11 ± 7.49** ^△△^	0.37 ± 0.11** ^△△^	655.79 ± 39.77** ^△△ ▲^
POL	200	319.07 ± 29.96** ^△△ ▲▲▽▽^	0.16 ± 0.10** ^△△ ▲▲▽^	641.93 ± 46.30** ^△△ ▲▲▽^

N: normal control group; M: model group; SASP: sulfamethoxypyridazine; CYN: ChangYanNing; POL: *Portulaca oleracea* L.; *p < 0.05 compared with the normal control group;

** *p* < 0.01 compared with the normal control group; ^△^ *p* < 0.05 compared with the model group;

△△ *p* < 0.01 compared with the model group;

▲ *p* < 0.05 compared with SASP;

▲▲ *p* < 0.01 compared with SASP; EGF: epidermal growth factor; MPO: myeloperoxidase; TNF-α: tumor necrosis factor-α.

Source: Elaborated by the authors.

### Effect of Portulaca oleracea L. on histological score of colon tissue in ulcerative colitis rats

**Figure 1:**
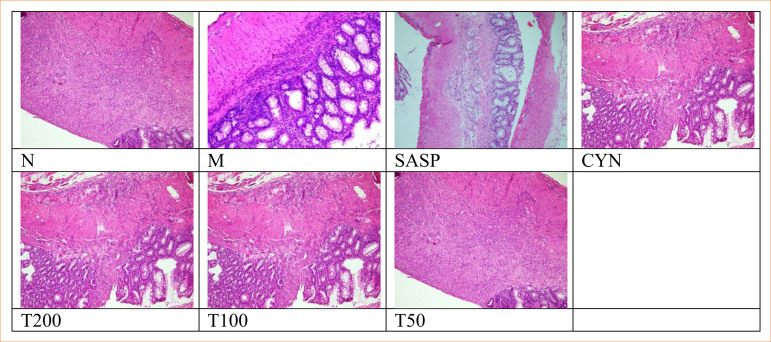
Colon tissue pathology of rats in each group (hematoxylin and eosin, ×200).

Source: Elaborated by the authors.

Compared to the control group, the epithelial cells, inflammatory cells, and total HS of rats in the model group showed a significant increase (*p* < 0.01). In comparison to the model group, the epithelial cells of rats in both the positive drug POL group and high-dose POL group exhibited a significant reduction in inflammatory cells and total HS (*p* < 0.01) (Table 8). The colon structure of the rats in the normal group appeared to be intact, with neatly arranged glands and normal crypts. The cell morphology was also observed to be normal, without any signs of congestion or edema, and no inflammatory cell infiltration was detected. In contrast, the rats in the model group exhibited a significant loss of goblet cells and crypt cells in the colon, along with a notable increase in inflammatory cell infiltration. However, each administration group showed varying degrees of goblet cell repair and reduced inflammatory cell infiltration (Fig. 1).

**Table 8:** Effect of *Portulaca oleracea* L. on histological score of ulcerative colitis rats (n = 10, mean ± standard deviation).

**Group**	**Dose (mg/kg)**	**Epithelial cells**	**Inflammatory cells**	**Total score for the HS**
N	-	1.0 ± 0.0	1.0 ± 0.0	2.0 ± 0.0
M	-	3.6 ± 0.5**	3.1 ± 0.6**	6.7 ± 0.9**
SASP	300	1.5 ± 0.5^△△^	2.1 ± 0.6**^△△^	3.6 ± 0.8**^△△^
CYN	300	2.6 ± 0.5**^△△^	3.1 ± 0.6**^▲▲^	5.7 ± 0.9**^▲▲^
POL	50	3.1 ± 1.1**	3.0 ± 1.0**^▲▲^	6.1 ± 2.1**^▲▲^
POL	100	2.3 ± 1.1**^△△▲^	2.9 ± 0.6**^▲^	5.2 ± 1.4**^▲▲^
POL	200	1.7 ± 1.2^△△^	1.5 ± 1.0^△△^	3.2 ± 1.5*^△△^

N: normal control group; M: model group; SASP: sulfamethoxypyridazine; CYN: ChangYanNing; POL: *Portulaca oleracea* L.;

* *p* < 0.05 compared with the normal control group;

** *p* < 0.01 compared with the normal control group; ^△^ *p* < 0.05 compared with the model group;

△△ *p* < 0.01 compared with the model group;

▲ *p* < 0.05 compared with SASP;

▲▲ *p* < 0.01 compared with SASP; HS: histological score.

Source: Elaborated by the authors.

### Effect of Portulaca oleracea L. on serum hemolysin production levels in ulcerative colitis rats

Compared to the control group, the serum hemolysin content of rats in the model group showed a significant reduction (*p* < 0.01). In comparison to the model group, each administration group exhibited a significant increase in serum hemolysin content (*p* < 0.01). Furthermore, the level of hemolysin production in each dose group of POL was comparable to that of the two positive drug groups (Table 9).

**Table 9:** Effect of *Portulaca oleracea* L. on serum hemolysin content in ulcerative colitis rats (n = 10, mean ± standard deviation).

Group	Dose (mg/kg)	Hemolysin expression level (HC50)
N	-	346.51 ± 5.65
M	-	316.72 ± 7.86**
SASP	300	341.31 ± 9.90^△△^
CYN	300	336.99 ± 10.06** ^△△^
POL	200	338.78 ± 8.12* ^△△^
POL	100	333.69 ± 8.15** ^△△^
POL	50	329.18 ± 10.37** ^△△^

N: normal control group; M: model group; SASP: sulfamethoxypyridazine; CYN: ChangYanNing; POL: Portulaca oleracea L.;

* *p* < 0.05 compared with the normal control group,

** *p* < 0.01 compared with the normal control group; ^△^ *p* < 0.05 compared with the model group;

△△ *p* < 0.01 compared with the model group.

Source: Elaborated by the authors.

### Effect of Portulaca oleracea L. on immunoglobulin IgG, IgM and IgA in serum of ulcerative colitis rats

Compared to the control group, the model group showed a significant reduction in immunoglobulin IgM (*p* < 0.01), and a significant increase in IgA and IgG levels (*p* < 0.01). Furthermore, the IgG and IgA levels in each dose group of POL were significantly lower than those in the model group (*p* < 0.01 or *p* < 0.05), while the IgM content was also significantly higher than that of the model group (*p* < 0.01). Additionally, there were no significant differences in the IgG and IgA levels between each dose group of POL and the two positive drug groups (Table 10).

**Table 10:** Effect of *Portulaca oleracea* L. on serum immunoglobulin IgG, IgM and IgA (n = 10, mean ± standard deviation).

Group	Dose (mg/kg)	IgG (mg/mL)	IgM (μg/mL)	IgA (mg/mL)
N	-	17.19 ± 3.96	749.22 ± 61.11	0.22 ± 0.10
M	-	23.85 ± 4.03**	378.76 ± 47.01**	1.05 ± 0.32**
SASP	300	20.48 ± 2.78^△△^	587.20 ± 28.78^△△^	0.36 ± 0.20^△△^
CYN	300	18.59 ± 1.88** ^△△^	685.47 ± 73.84^△△^	0.35 ± 0.14^△△^
POL	200	19.53 ± 2.39^△△^	725.21 ± 51.22^△△ ▲^	0.50 ± 0.27** ^△△^
POL	100	20.77 ± 3.32* ^△^	656.04 ± 25.53^△△^	0.41 ± 0.33^△△^
POL	50	20.42 ± 2.72* ^△^	701.59 ± 20.60^△△^	0.28 ± 0.12^△△^

N: normal control group; M: model group; SASP: sulfamethoxypyridazine; CYN: ChangYanNing; POL: *Portulaca olerace*a L.;

* *p* < 0.05 compared with the normal control group,

** *p* < 0.01 compared with the normal control group;

△ *p* < 0.05 compared with the model group;

△△ *p* < 0.01 compared with the model group;

▲ *p* < 0.05 compared with SASP.

Source: Elaborated by the authors.

### Effect of Portulaca oleracea L. on serum complement C3 and C4 contents in ulcerative colitis rats

Compared to the control group, the levels of C3 and C4 in the serum of rats in the model group showed a significant increase (*p* < 0.01 or *p* < 0.05). In comparison to the model group, all groups had a significant reduction in serum C3 levels (*p* < 0.01). Additionally, there was a significant decrease in serum C4 levels in the positive drug group (*p* < 0.05) (Table 11).

**Table 11:** Effect of *Portulaca oleracea* L. on serum complement C3 and C4 in ulcerative colitis rats (n = 10, mean ± standard deviation).

**Group**	**Dose (mg/kg)**	**C3 (ng/mL)**	**C4 (ng/L)**
N	-	839.16 ± 60.22	312.51 ± 73.64
M	-	1606.51 ± 105.41**	398.98 ± 131.60*
SASP	300	1143.45 ± 125.69**^△△^	304.33 ± 58.41^△^
CYN	300	1225.10 ± 107.53**^△△^	324.42 ± 33.85
POL	200	1273.87 ± 130.33**^△△^	329.46 ± 69.44
POL	100	1322.21 ± 131.67**^△△^	337.56 ± 59.32
POL	50	1342.40 ± 108.64**^△△▲^	382.33 ± 124.32

N: normal control group; M: model group; SASP: sulfamethoxypyridazine; CYN: ChangYanNing; POL: *Portulaca oleracea* L.;

* *p* < 0.05 compared with the normal control group,

** *p* < 0.01 compared with the normal control group;

△ *p* < 0.05 compared with the model group;

△△ *p* < 0.01 compared with the model group;

▲ *p* < 0.05 compared with SASP.

Source: Elaborated by the authors.

### Effect of Portulaca oleracea L. on splenocyte T lymphocyte subsets in ulcerative colitis rats

**Figure 2:**
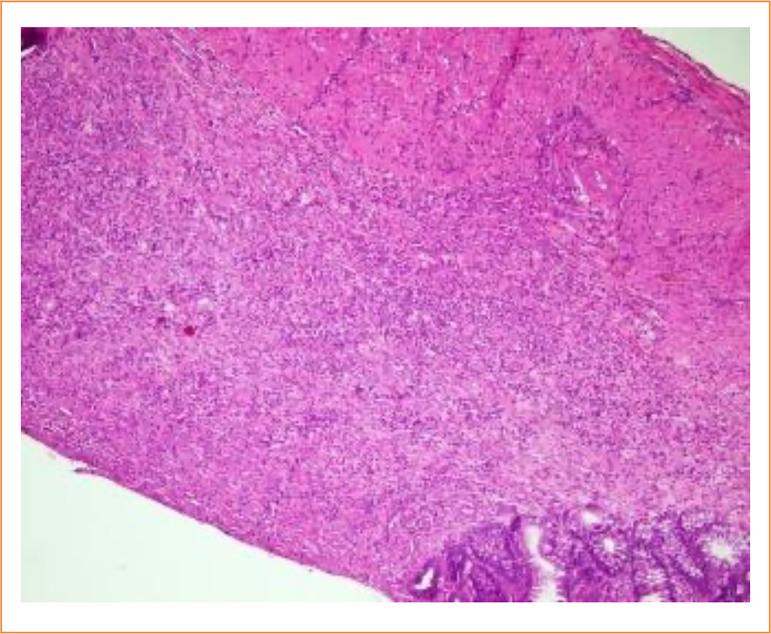
Effect of *Portulaca oleracea* L. on Th17 cells of spleen cells in ulcerative colitis rats.

Source: Elaborated by the authors.

During FACS Calibur detection, the CD3+T lymphocyte population was specifically chosen for analysis of CD4, CD8, and CD25 staining. Additionally, the CD3+CD4+T lymphocyte population was selected to analyze interleukin (IL)-17A + Th17 cells. The results of flow cytometry revealed a significant increase in the proportion of CD3+/CD4+ and Th17 cell subsets, as well as CD4+/CD8+ in the spleen of rats in the model group compared to those in the normal group (*p* < 0.01). Conversely, this proportion was significantly lower than that of the normal group (*p* < 0.01). Furthermore, when compared with the model group, it was observed that the cell levels and proportions of each administration group recovered to varying degrees (Table 12 and Fig. 2).

**Table 12:** Effect of POL on splenocyte T lymphocyte subsets in UC rats ( ).

Group	Dose (mg/kg)	CD3+ (%)	CD3+CD4+ (%)	CD3+CD8+ (%)	CD4+/CD8+	CD4+CD25+/CD4+	IL-17+CD4+/CD4+
N	-	30.53 ± 1.58	60.66 ± 4.35	28.80 ± 3.54	2.34 ± 0.28	0.16 ± 0.02	0.11 ± 0.01
M	-	24.91 ± 1.49**	73.86 ± 3.76**	24.90 ± 5.12*	3.34 ± 0.47**	0.11 ± 0.02**	0.17 ± 0.02**
SASP	300	28.10 ± 2.26^△^	66.63 ± 3.77**	28.46 ± 3.57^△^	2.60 ± 0.26^△△^	0.14 ± 0.01^△△^	0.14 ± 0.01** ^△△^
CYN	300	29.16 ± 4.65^△△^	66.92 ± 4.46** ^△△^	28.39 ± 3.11^△^	2.60 ± 0.23^△△^	0.15 ± 0.02^△△^	0.15 ± 0.02**
POL	200	27.97 ± 3.45^△^	65.06 ± 3.83* ^△△^	28.76 ± 3.23^△^	2.50 ± 0.29^△△^	0.15 ± 0.02^△△^	0.11 ± 0.01** ^△△^
POL	100	27.39 ± 3.60*	64.91 ± 5.37* ^△△^	26.36 ± 1.98	2.72 ± 0.30** ^△△^	0.11 ± 0.01**	0.15 ± 0.02** ^△^
POL	50	27.97 ± 3.45^△^	66.16 ± 2.35* ^△△^	25.60 ± 1.22	2.84 ± 0.13** ^△△^	0.13 ± 0.02** ^△^	0.17 ± 0.02** ^▲▲^

N: normal control group; M: model group; SASP: sulfamethoxypyridazine; CYN: ChangYanNing; POL: *Portulaca oleracea* L.;

* *p* < 0.05 compared with the normal control group,

** *p* < 0.01 compared with the normal control group;

△ *p* < 0.05 compared with the model group;

△△ *p* < 0.01 compared with the model group;

▲▲ *p* < 0.01 compared with SASP.

Source: Elaborated by the authors.

## Discussion

The model of chronic UC induced by allogeneic immunity combined with acetic acid closely resembles the course and mechanism of human UC in terms of disease progression and inflammation. In comparison to other UC models, it is characterized by a prolonged disease course, stability, reliability, and a high success rate^20,21^. Freund’s complete adjuvant is able to enhance the antibody response to the antigen and stimulate local immune response. Additionally, acetic acid is capable of causing damage to the intestinal mucosa, increasing vascular permeability, initiating inflammation, and ultimately inducing a chronic UC model in rats^22^.

In this experiment, following the oral administration of cockroach POL extract, the relevant indicators of UC rats showed varying degrees of improvement. IL-4 is a cytokine secreted by type II helper T cells, and mast cells can also secrete this protein. This factor participates in humoral immunity by regulating antibody class switching to IgE and induces the differentiation of CD4 T cells into type II helper T cells. IFN-γ is an inflammatory mediator produced by lymphocytes. Both IFN-γ and IL-4 play opposing yet crucial roles in maintaining intestinal immune balance. Clinical findings have revealed a decrease in the expression of IL-4 during the remission period of UC patients, while the expression of IFN-γ increases. In the model group, there were a significant reduction in IL-4 content and a notable increase in IFN-γ content, consistent with the clinical symptoms observed in UC patients during remission^23^. IL-8, TNF-α, and MPO have been shown to enhance lysosomal enzyme activity and phagocytosis of neutrophils, while also generating a substantial number of toxic substances that exacerbate intestinal inflammation.

The observed trends were consistent with the clinical symptoms experienced by patients with UC during remission^24^. IL-17 is primarily secreted by CD4+ T cells and exhibits potent pro-inflammatory activity. It serves as a crucial soluble factor in the process of T cell induction and the promotion of inflammation, exacerbating and hastening intestinal inflammation. EGF, a polypeptide functional growth factor, plays a significant role in both the damage and repair of the intestinal mucosa^25,26^. Both CRP and IgG are proteins that sharply increase in levels when the body is infected or tissue is damaged^26^.

The experimental results demonstrated a significant increase in the levels of IL-4 and EGF in rats in the high-dose POL group and the positive drug group, while there was a notable decrease in the levels of IL-8, IL-17, IFN-γ, CRP, IgG, TNF-α, and MPO. Furthermore, it is suggested that cockroach POL extract has a certain therapeutic effect on UC.

Changes in T cell subsets in rats revealed that one of the causes of ulcerative colitis may be provoked by dysfunction of T lymphocytes, in which intestinal damage caused by CD4+ T lymphocytes is characteristic and recurrent. The Th1 pathway can induce delayed-type hypersensitivity reactions and macrophage activation, both of which are associated with the activation of CD4+ T lymphocytes. The proliferation of B lymphocytes can lead to a humoral immune response, which is linked to the cellular immune response or through the Th2 pathway, ultimately resulting in colon mucosal damage and an inflammatory response^27^. Abnormal changes in the CD4+/CD8+ ratio may be attributed to cellular immune damage or an inflammatory response, serving as a significant indicator of the body’s immune regulation balance. Under normal circumstances, these two components maintain a specific ratio to uphold the body’s cellular immune function. An imbalance in this proportion can lead to compromised immune function^28^. Regulatory T cells play a crucial role in maintaining the body’s immune balance and peripheral immune tolerance. This subset of T cells exerts negative immune regulatory effects^29^.

Initial research has shown that there is a decrease in CD4+CD25+ cells in patients with active UC, suggesting a positive correlation between the expression rate of CD4+CD25+ cells and immunity^30^. Animal experiments have demonstrated that the dysregulation of Th17/Treg (regulatory T cells) differentiation is linked to the onset of inflammatory bowel disease^31^. Concurrently, there are studies suggesting that Th17 cell factors may play a role in the modulation of inflammation^32^.

From the results of this experiment, the expression of CD3+ in the model group was significantly reduced, while there was a significant increase in CD4+T and IL-17+CD4+T cells. Additionally, there were a decreasing trend in CD8+ expression, an upward trend in the CD4+/CD8+ ratio, and a decrease in CD4+CD25+/CD4+ expression. The POL drug group demonstrated varying degrees of increased CD3+ expression, reduced levels of IL-17+CD4+T cells and the CD4+/CD8+ ratio, as well as enhanced CD4 +CD25+/CD4 +expression. This suggests that the POL drug can regulate the balance of T lymphocyte subpopulations and reduce the risk of immune response to achieve a reduction in inflammatory damage. The process of complement activation can generate active fragments such as C3, C4, and other inflammatory mediators, leading to the production of many immune complexes (Ig) and resulting in mucosal congestion, edema, ulcers, etc.

The findings from this experiment demonstrated a significant increase in the levels of complement C3 and C4 in the serum of rats in the model group. Additionally, there was a notable increase in the levels of IgG and IgA, indicating activation of complement, production of immune complexes, B cell activation, and exacerbation of inflammatory response.

## Conclusion

The POL extracted has been found to have a therapeutic effect on UC induced by allogeneic antigen combined with acetic acid in rats. It can promote the repair of intestinal mucosa, inhibit inflammation in the body, and improve the pathological changes of colon tissue. The therapeutic mechanism of this treatment may be associated with the inhibition of pro-inflammatory factors such as IL-8, IL-17, IFN-γ, CRP, IgG, TNF-α and MPO, while also up-regulating the expression of IL-4 and EGF. In addition, it also regulates the balance of T lymphocyte subsets by modulating the expression of CD3+, CD4+, CD8+, and IL-17+. Furthermore, it upregulates the contents of hemolysin and IgM, while downregulating the contents of IgG, IgA, complement C3 and C4. These two aspects work synergistically to regulate together and ultimately achieve the purpose of treating UC.

## Data Availability

Data will be available upon request.
